# Perovskite/Silicon Tandem Solar Cells Above 30% Conversion Efficiency on Submicron-Sized Textured Czochralski-Silicon Bottom Cells with Improved Hole-Transport Layers

**DOI:** 10.1021/acsami.4c09264

**Published:** 2024-10-30

**Authors:** Angelika Harter, Kerem Artuk, Florian Mathies, Orestis Karalis, Hannes Hempel, Amran Al-Ashouri, Steve Albrecht, Rutger Schlatmann, Christophe Ballif, Bernd Stannowski, Christian M. Wolff

**Affiliations:** † Solar Energy Department, Helmholtz Zentrum Berlin (HZB), Schwarzschildstraße 3, 12489 Berlin, Germany; ‡ Institute of Electrical and Microengineering (IEM), Photovoltaics and Thin-Film Electronics Laboratory (PV-Lab), École Polytechnique Fédérale de Lausanne (EPFL), Rue de la Maladière 71b, 2000 Neuchâtel, Switzerland; § CSEM, Sustainable Energy Center, Rue Jaquet-Droz 1, 2000 Neuchâtel, Switzerland

**Keywords:** perovskite/silicon tandem solar cells, self-assembled monolayer, submicron-sized texture, co-assembly, photovoltaics

## Abstract

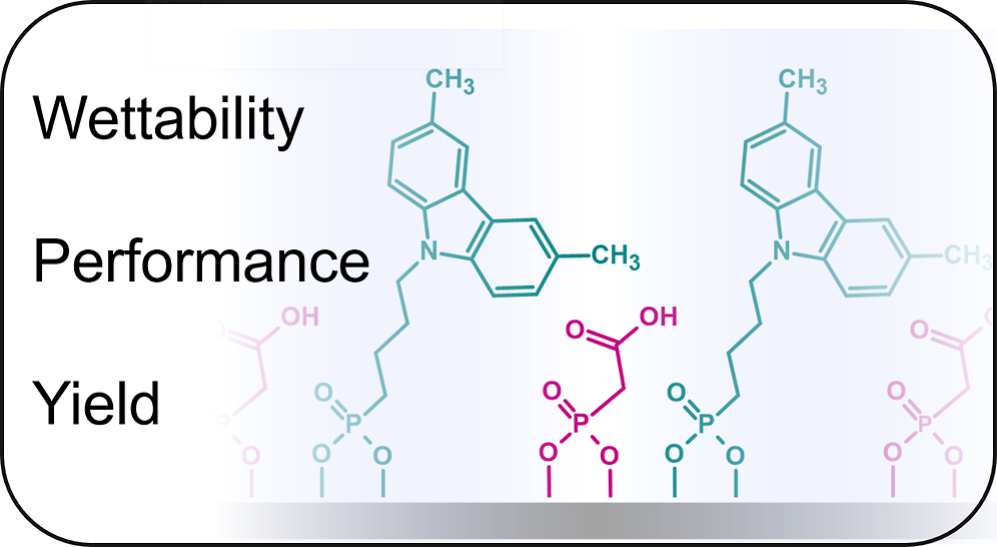

In perovskite/silicon tandem solar cells, the utilization of silicon heterojunction (SHJ) solar cells as bottom cells is one of the most promising concepts. Here, we present optimization strategies for the top cell processing and their integration into SHJ bottom cells based on industrial Czochralski (Cz)-Si wafers of 140 μm thickness. We show that combining the self-assembled monolayer [4-(3,6-dimethyl-9*H*-carbazol-9-yl)butyl]phosphonic acid (Me-4PACz) with an additional phosphonic acid (PA) with different functional groups, can improve film formation when used as a hole transport layer improving wettability, minimizing shunt fraction and reducing nonradiative losses at the buried interface. Transient surface photovoltage and transient photoluminescence measurements confirm that the combined Me-4PACz/PA layer has similar charge transport properties to Me-4PACz alone. Moreover, this work demonstrates the potential for thin, double-side submicron-sized textured industry-relevant silicon bottom cells yielding a high accumulated short-circuit current density of 40.2 mA/cm^2^ and reaching a stabilized power conversion efficiency of >30%. This work paves the way toward industry-compatible, highly efficient tandem cells based on a production-compatible SHJ bottom cell.

## 1 Introduction

Silicon heterojunction (SHJ) solar cells in perovskite/silicon tandem solar cells are one of the most promising concepts as bottom cells achieving world records. Champion devices mostly use a thick float zone (FZ) bottom cell, which is not economically optimal for industrial mass production. These high-efficiency tandem solar cells come as state-of-the-art with a polished front and textured rear silicon bottom cell.^1,2^ A flat interface between the two subcells leads to reflection losses at the interface and, additionally, is challenging to realize in industry. Driven by resource efficiency and cost reduction, Czochralski (Cz)-grown SHJ bottom cells with a thickness of 100–150 μm and double-sided micro textured surfaces are standard in industrial fabrication. To further improve the light in-coupling while still meeting the requirements for industrial viable perovskite/silicon tandem solar cells, different approaches such as double-sided texturing of the bottom cell, interface engineering, or improving the performance of individual layers are widely discussed.^3^ The concept of double-sided textured bottom cells is a key adaptation in the design of the current champion devices, such as those from the King Abdullah University of Science and Technology, which achieve a power conversion efficiency (PCE) of 33.7%^4^ and LONGi, who holds the world record with an outstanding PCE of 33.9% and recently 34.6% on Cz-silicon (Cz-Si) bottom cells.^5^

In monolithic perovskite/silicon tandem solar cells, different approaches are used to fabricate the overlying perovskite films on c-Si bottom cells. A conformally covered pyramidal texture can be realized by co-evaporation of the perovskite absorber with low reflection losses in both subcells and high stability.^6^ Following a hybrid two-step deposition method combining thermal evaporation and spin coating, a conformal and uniform coverage of the perovskite absorber on micrometric pyramids has currently been shown to achieve a PCE of 31.25%.^7^ In contrast, spin coating of the perovskite following the simple, low-cost solution-processing route results in a nonconformal but planarized surface of the perovskite surface.^8,9^ Solution-processing of the perovskite absorber on the standard micrometer-sized textures remains challenging, resulting in shunting of the top cell. Therefore, double-side submicron-sized textured bottom cells have become an intriguing approach to improve light in-coupling while still allowing solution-processing of high-quality perovskite absorbers that meet the requirements for industry production-line compatible perovskite/silicon tandem solar cells. Considering the near-infrared loss, the combination of thin Cz-Si bottom cells in perovskite/silicon tandem solar cells remains a challenge for high performance.

Liu *et al*.^10^ demonstrated a PCE of 30.5% on a submicron-sized textured FZ-Si bottom cell (∼260 μm thickness), and using a SHJ bottom with a sinusoidal submicron-sized texture (300 nm in height) obtained by nanoimprinting, Tockhorn *et al*.^11^ showed an enhanced optical device performance with a reduction in reflectance loss of about 0.5 mA/cm^2^ compared to a front-polished bottom cell. Moreover, the device yield was improved due to the submicron texture. This strengthens the motivation to replace the front polished cells with double-side submicron-textured bottom cells. Recently, an increasing number of groups have also been working on industrially relevant SHJ solar cells based on Cz-Si. Mao *et al*.^12^ reported a PCE of 28.84% on a 150 μm thick Cz-Si bottom cell with a hybrid vapor/solution processed top cell. Demonstrating their high potential compared to FZ material, Köhnen *et al*.^13^ showed comparable PCE of monolithic perovskite/silicon tandem solar cells based on a 100 μm thick Cz-Si bottom cell and a ∼300 μm thick FZ-Si bottom cell. Reducing the thickness of the bottom cells led to a decrease in the current density, resulting in an accumulated current density of 37.37 mA/cm^2^. A PCE of 29.2% was reported by Yamamoto *et al*.^14^ based on a 145 μm thick Cz-Si wafer, achieving an accumulated current density of 39.3 mA/cm^2^. The *JV* characteristics of these cells are given in the Supporting Information Table S1. The use of industrial Cz-Si wafer material is also crucial for upscaling and has been successfully demonstrated by different working groups.^15,16^

As mentioned above, another approach to improve optoelectronic properties is interface engineering. Several approaches have been shown to further improve the top cell, one of which is the work on the hole transport layer (HTL) sandwiched between the indium thin oxide (ITO) and the perovskite layer. Various materials are used for the HTL, such as PEDOT: PSS, poly(triarylamine) (PTAA), nickel oxide (NiO_*x*_)^17^ or self-assembling monolayers (SAMs).^18,19^ As an alternative to other inorganic HTLs, NiO_*x*_ has been shown to increase both current density and open circuit voltage with improved stability.^17^ Effective interfacial passivation and fast hole extraction can be realized when using SAMs in perovskite/silicon tandem solar cells, resulting in high efficiency with low nonradiative losses and simple processing.^19^ With this SAM-based approach, parasitic absorption is kept at a minimum, and simple process control is maintained. Moreover, material consumption is low, which again is important for industrialization. These SAMs, have been shown to improve quasi-Fermi-level splitting (QFLS) compared to e.g., PTAA by reducing nonradiative recombination at the perovskite/HTL interface.^19^ Showing fast hole extraction and allowing high QFLS, Me-4PACz ([4-(3,6-dimethyl-9*H*-carbazol-9-yl)butyl]phosphonic acid) is one of the most promising HTLs widely used in highly efficient and stable perovskite/silicon tandem solar cells.^18^ However, Me-4PACz is often associated with limited wetting, rendering perovskite films with macroscopic holes on planar substrates.^11^ The addition of a small molecule to the commonly used SAMs, e.g., also with a PA anchor group, can contribute to an improved wetting of the SAM and at the same time improve the performance, including on rough substrates. Optimized surface functionalization and interaction with the perovskite layer in perovskite solar cells has been achieved by combining two molecules,^20^ a hole-selective SAM with an alkyl ammonium salt, resulting in coassembled monolayers.^21^ The alloying of Me-4PACz with phosphorylcholine chloride has been shown to result in an improved monolayer coverage,^22^ as has the addition of 3-MPA to the SAM.^23^ Adding the commercially available 1,6-hexylenediphosphonic acid (6dPA) to the Me-4PACz precursor solution has been shown to improve the wetting and therefore device yield, while resulting in a very similar performance.^24^

In this work, we present tandem cells on SHJ bottom cells textured by wet-etching random pyramids (∼600 nm average height). We have previously shown that the pyramid height can be adjusted by alkaline texturing with no current loss in SHJ cells compared to our standard (micron-sized) textured surfaces (see Figure S1).^8^ To achieve high performance shunt-free perovskite/silicon tandem solar cells with solution-processed with high yield top cells, the perovskite absorber thickness must be thicker than the pyramid texture height. Since high quality wide band gap perovskite absorbers are typically 600–800 nm thick and do not usually conformally cover the surface when processed from solution, the pyramid height must be adjusted accordingly. Thicker layers are challenging to process, due to the solubility limit^25^ of the perovskite precursors in the common solvent systems (e.g., DMF/DMSO) and the fast crystallization dynamics resulting in a rough, wrinkled morphology. As a result, thicker (>1 μm) perovskite films suffer from poor carrier collection due to insufficient diffusion lengths.^26^ In addition, a rough morphology makes the processing, surface treatment, and interface engineering atop more difficult, and from a different microstructure than the thinner counterparts. Additionally, thickening the absorber can result in increased material costs.^27^

Moreover, this work aims to improve the wetting of Me-4PACzs as HTL. Therefore, different molecules with a PA anchoring group were combined with the Me-4PACz, and their impact on film formation and charge extraction was investigated in detail. Based on the results of this work, a perovskite/silicon tandem solar cell with a PCE > 30% is demonstrated, highlighting the potential of 140 μm thin silicon bottom cells for industry-compatible, highly efficient tandem cells.

## 2 Results

### 2.1 Wetting Improvement

**Figure 1:**
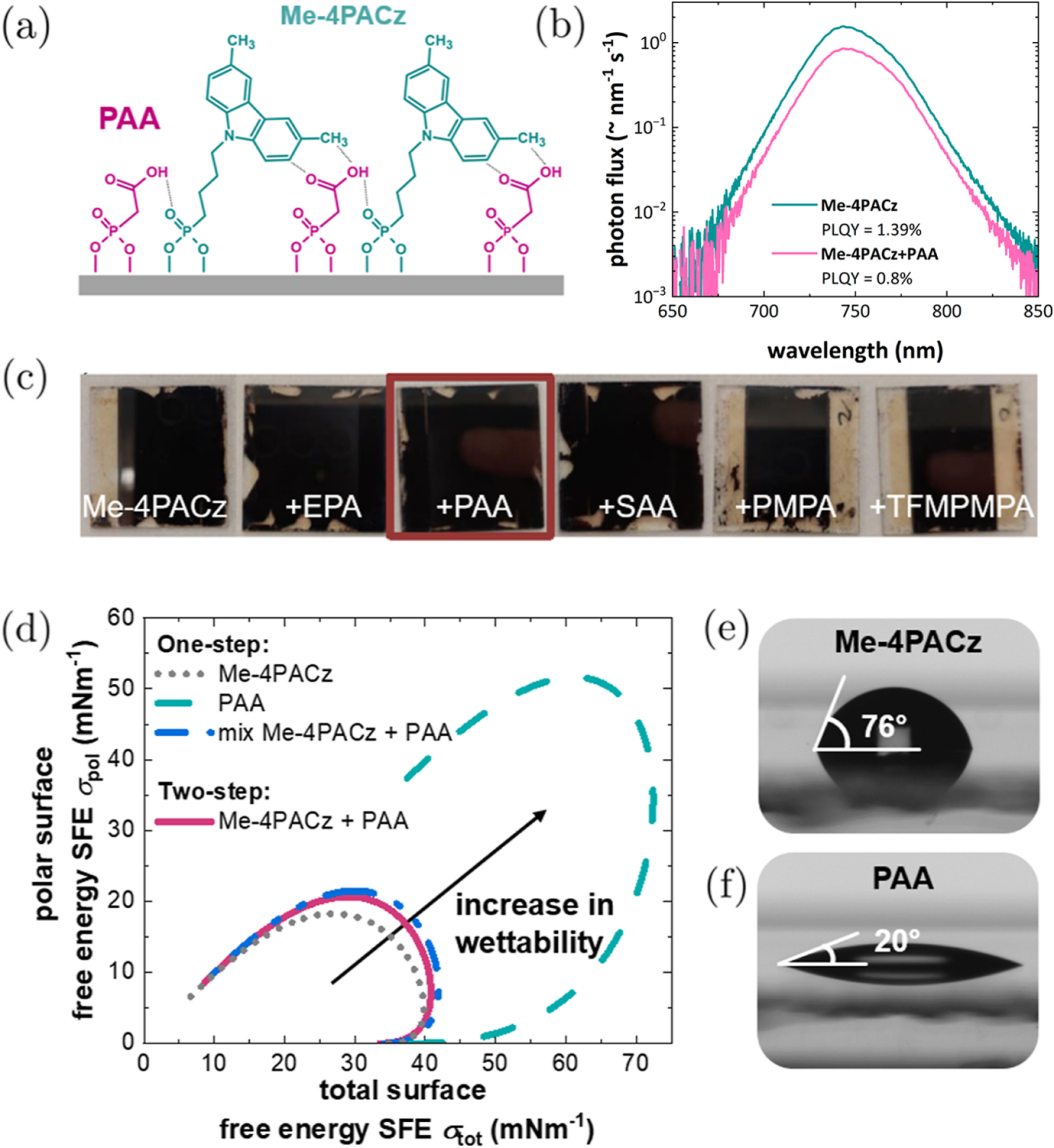
(a) Schematic drawing of the coadsorption showing the potential arrangement of the Me-4PACz molecules and phosphonoacetic acid (PAA), emphasizing a potentially enhanced dipole–dipole interaction (hydrogen bridge bond); (b) photoluminescence quantum yield (PLQY) measurements of Me-4PACz and the sequential combination of Me-4PACz with PAA measured on a glass/ITO/HTL/perovskite stack; (c) wetting characteristics of the HTL mixtures with Me-4PACz and ethyl PA (EPA), PAA, 3-sulfoacetic acid (3-SAA), 4-pyrindynylmethyl PA (PMPA), and {[3-(trifluoromethyl)phenyl]methyl}phosphonic acid (TFMPMPA)—photographs of spin-coated perovskite films on different treated HTMs to visualize the wetting behavior of Me-4PACz and different small PAs in combination with Me-4PACz on ITO/glass substrates; (d) wetting envelope with the surface free energy (SFE) measured on glass/ITO/HTL samples using Me-4PACz, PAA, and the two-step approach as HTL; (e) contact angle of water on glass/ITO/Me-4PACz sample; (f) contact angle of water on glass/ITO/PAA sample.

To improve the film coverage when using Me-4PACz as a hole-selective material under the perovskite, the co-adsorption of SAMs and smaller molecules with a PA anchoring group having different functional groups was studied. The performance of the HTL and its coverage characteristics depend strongly on different properties such as the orientation of the PA the intermolecular space, the aggregation, and the underlying intermolecular forces, such as dipole–dipole interactions. Th e aim of this work was to combine the Me-4PACz molecules with an additional smaller PA, which could either fill uncovered areas or form a wetting overlayer, increasing the polarity and improving the film coverage of the perovskite absorber, as illustrated in Figure 1a. These additional PAs also allow the formation of more dipole–dipole interactions through hydrogen bridge bonding to adjacent phosphonic acid anchor groups.

Initially, solutions of different functionalized PAs and a sulfonic acid mixed with Me-4PACz were investigated with respect to wettability and perovskite quality (see Figure 1). The different PAs were diluted in ethanol (EtOH) and mixed with Me-4PACz (in EtOH) in a ratio of 1:4 and spin-coated on glass/ITO samples. The nonradiative recombination losses *q*·*V*_nonrad_ extracted by steady-state PLQY measurements of perovskite films before and after C_60_ deposition are shown in Figure S2. The PL spectra are shown in Figure 1b for Me-4PACz and the sequential application of Me-4PACz and PAA, showing a slightly increased PLQY when using Me-4PACz alone. Improved film coverage was observed by adding EPA, PAA, and 3-SAA during HTL processing, as depicted in Figure 1c. Using 4-pyridinylmethyl PA (PMPA) and {[3-(trifluoromethyl)phenyl]methyl}phosphonic acid (TFMPMPA), a reduction in nonradiative losses was measured. However, the wetting limitation remained. 3-SAA led to an increase in nonradiative losses, whereas both EPA and PAA yield nonradiative losses in the same range as the Me-4PACz reference, but improved the film coverage. Moreover, it has been shown that PAs with carboxyl acid termination [e.g., phosphonopropionic acid (PPA)] can significantly improve the stability of the device,^28^ which is why we continued our investigations with PAA.

To further optimize and understand the effect of mixed PAs, different methods for their combination with the large Me-4PACz molecules were investigated, such as mixing with Me-4PACz (ratio 1:4) or sequential spin coating (see Figures S3 and S4). Sequential deposition with an intermediate annealing step resulted in similar PLQY as pure Me-4PACz films. Therefore, this method (spin-anneal-spin-anneal) was used in the following experiments. With the focus now on PAA, the impact of the spacer between the functional group and the anchoring group, that is, the nonpolar carbon chain, was additionally investigated. It has been shown that the spacer group of a SAM has a large influence, not only on the wetting characteristics but also on the hole transfer by tunneling through the spacer group and the dipole moment.^29^ For that, molecules with a longer alkyl chain were used, namely 3-PPA and 3-phosphonohexanoic acid (PHA). Solutions of the molecules PAA, PPA, and PHA were prepared in EtOH (1 mg/mL). The HTL was formed in a two-step approach: spin-coating of the large Me-4PACz molecule, annealing of the layer, followed by spin-coating and annealing of the small PA. Further investigations of the spin-coating sequence, e.g., mixing the small PA directly with the Me-4PACz solution (1 mg/mL), and variations of the two-step approach (washing in between, no annealing step in between) are shown in Figure S3 in the Supporting Information. This multi-step approach adds control to the formation and infiltration of the coadsorbed SAM-in-fillers. The annealing step was conducted at 100 °C for 10 min. The QFLS was unaffected by spin-coating either Me-4PACz alone or spin-coating Me-4PACz with PAA (Figure S4). When spin-coated with PAA alone (without Me-4PACz), there was a decrease of ∼10 mV compared to the Me-4PACz reference, indicating inferior interface/bulk absorber quality. When PPA or PHA was added instead of PAA, a ∼16 meV increase of the QFLS was observed compared to the Me-4PACz-only reference—Figure S4. The improvement in QFLS shows that the perovskite absorber grown on top of Me-4PACz +PPA (or +PHA)HTLs has lower nonradiative recombination losses, which can be explained by an improved buried interface quality or by a different absorber bulk/top surface quality. The addition of a fluorinated PA to the perovskite precursor—for improved perovskite/C_60_ interface passivation—results in an average increase of ∼30 meV, as shown in our previous work^2^ and is confirmed also for these multi-step formed HTL (Figure S4).

To quantify the wettability, the surface free energy (SFE) of the different surfaces is calculated using the method of Owens–Wendt–Raebel–Kaelble.^30,31^ The wettability of the substrate is illustrated by the wetting envelope, shown in Figure 1d. Any ink inside the envelope will wet the substrate and any ink outside the envelope will not wet the surface properly; therefore, at higher surface tensions it will not wet the surface properly. To quantify wettability, the SFE measurement, also known as the sessile drop method, is a useful tool. For that, the contact angles of diiodomethane (hydrophobic) and water with a known dispersive and polar surface tensions were measured separately. Using Young’s equation,^32^ the wetting behavior of a liquid on the substrate can be expressed as follows.
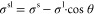
1where *θ* = contact angle, *σ*^sl^ = interfacial surface energy, *σ*^s^ = solid surface energy, and *σ*^l^ = liquid surface tension, of which the latter two can be split into their polar and dispersive parts using the method of Owens–Wendt–Raebel–Kaelble, resulting in an implicit formula given by eq 2
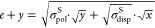
2where *x* = σ_disp_^l^, y = σ_pol_^l^. Using eq 2, the wettability of the substrate can be illustrated by the wetting envelope, shown in Figure 1d, where any solvent inside the curve will wet the substrate, and any solvent outside, i.e., with a higher surface tension, will not wet the surface. Me-4PACz coated glass/ITO substrates show a significantly lower wettability compared to PAA, as shown by the smaller wetting envelope in Figure 1d. Using PAA alone, the SFE increases from 40 to 72 mN m^–1^ compared to Me-4PACz (Table S2). This is not due to a change in the dispersed fraction (which remains roughly the same for all sequences (Table S3) and the Me-4PACz reference), but due to an increase in the polar fraction by a factor of 6.9 (Table S4). Thus, the combination of PAA (or PPA, PHA) with Me-4PACz leads to an increased surface polarity and improves the wettability of the surface. The wetting envelope of the different fillers and varying deposition methods can be found in Figure S5. When PAA is spin-coated in a two-step approach without the intermediate annealing step, a strongly increased polarity is observed (Table S4). We hypothesize that a substantial fraction of the Me-4PACz molecules is displaced when the small PA is spin-coated onto Me-4PACz before annealing. Therefore, a higher amount of PA molecules—relative to Me-4PACz—covers the glass/ITO surface. An annealing step between the two Me-4PACz and PAA spin-coating steps promotes Me-4PACz molecules to bind firmly to the ITO, making subsequent displacement or mixing much less likely, and only a small change in the SFE is observed. Likewise, a washing step after annealing of Me-4PACz shows no further impact on the wetting as Me-4PACz binds strongly to the ITO surface (Table S4). While there is no strong difference between PAA and PPA, a further increase in chain length for PHA leads to a higher SFE. Spin-coating first the small filler molecule and then Me-4PACz, leads to an overall increase in SFE due to a strong increase in polarity (Tables S2 and S4). Using PAA can enable the appearance of keto-enol tautomerism (carbonyl double bond breaks while an alkene double bond is formed, stabilized by water bridge interaction), resulting in lower flexibility and fewer dipole–dipole interactions.

This increase in polarity of the underlying SAM layer leads to an improvement in wetting with triple-cation perovskite [3CAT; Cs_0.05_(FA_0.90_MA_0.10_)_0.95_Pb(I_0.80_Br_0.20_)_3_] as the absorber. This becomes even more crucial when using pentafluorobenzylphosphonic acid (pFBPA) as an additive in the perovskite solution,^2^ as it not only increases the SFE (compare Figure S5) but is also more compatible with more polar HTLs.

### 2.2 Performance of Single Junction Solar Cells

**Figure 2:**
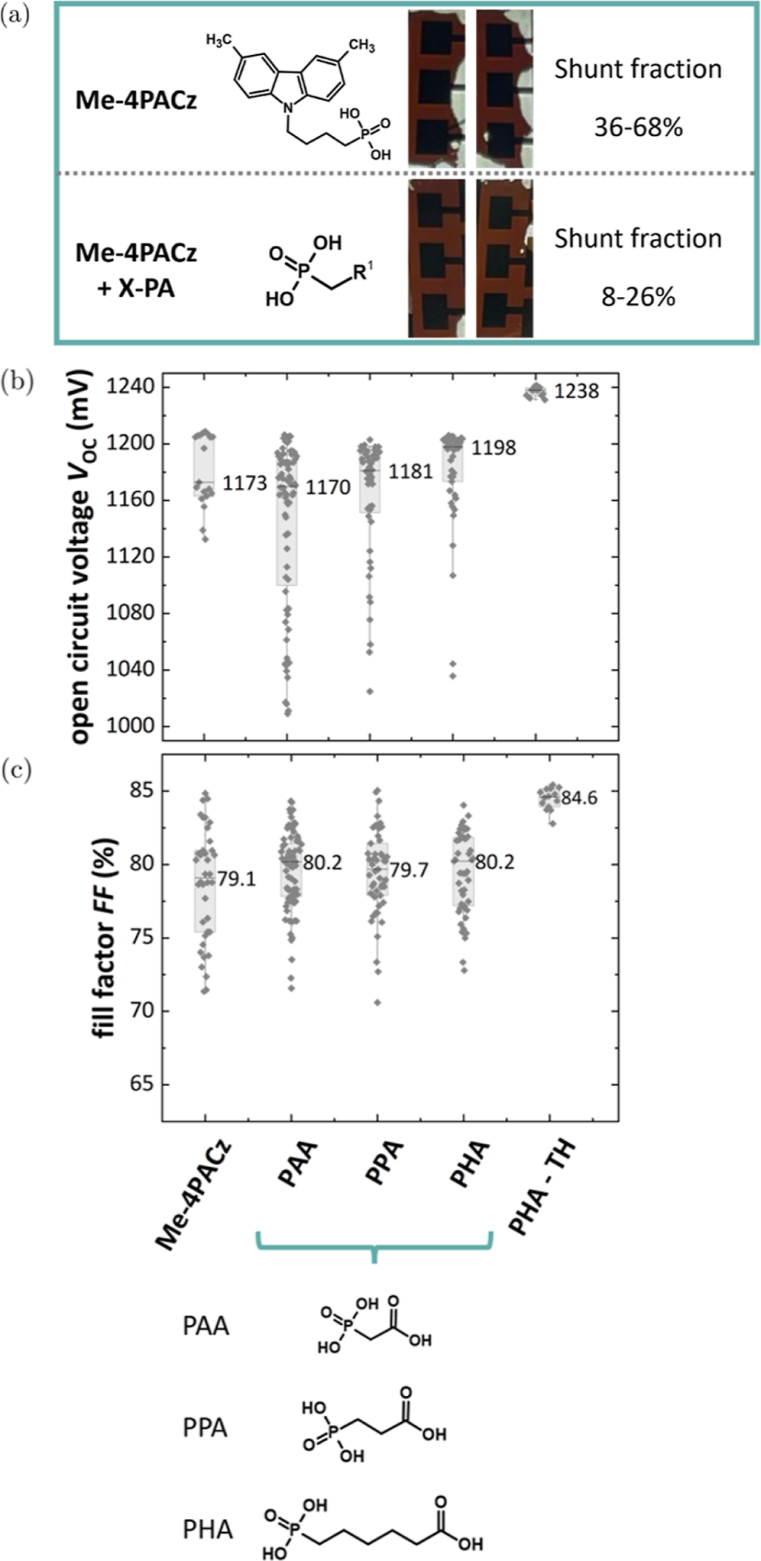
(a) Coverage of perovskite layers shown by close-up images using Me-4PACz and the combination with X-PA (here PAA), which shows a 2–5 times lower shunt probability. Single junction solar cell performance. (b) *V*_OC_ for the two-step approach of Me-4PACz and Me-4PACz + PAA, +PPA, and +PHA, and +PHA + TH (PHA with triple halide absorbers) and (c) FF.

The impact of using small PAs and different chain lengths of the spacer group of the small PA on the device performance in single junction perovskite solar cells is shown in Figure 2b,c. By using the small PAs, there is a strong yield improvement (compare Figure 2a) due to a strongly reduced shunt fraction (Figure 2b,c). Yield improvement leads to higher average FFs and *V*_OC_s with the small PAs compared to the Me-4PACz alone configuration. The increasing chain length (PAA–PPA–PHA) also corresponded to a slightly increased average *V*_OC_ of 1.198 V and comparable FFs. While the peak *V*_OC_ obtained using PAA and PPA decreased by 20 mV compared to Me-4PACz alone, using PHA shows no discrepancy. Over two batches, Me-4PACz-based cells had a shunt fraction (percentage of cells with a *V*_OC_ ≪ 1 V, indicating shunted cells) of 36%, while PPA, PHA, and PAA ended up at 25%, 25%, and 10%, respectively. As there were additional experiments carried out with Me-4PACz and PAA, the shunt fraction increased to 68% for Me-4PACz, while it decreased to 8% for PAA, emphasizing that the yield in working devices increases strongly when using these fillers (see Figure 2a). The influence on *J*_SC_ (no statistically relevant effect observed) and PCE, as well as their respective external quantum efficiencies (EQEs), are shown in Figure S6.

In order to maintain the improved FF, the spin-coating sequence - yet agin - must be considered. We observed the best wetting when the PA (PAA) was spin-coated directly after Me-4PACz, and a higher FF and efficiency are reached when annealing in between, which also improves the wetting due to its increased polar character. Moreover, a sequential application is more promising than adding the small molecule directly to the solution of the Me-4PACz solution, resulting in both higher FF and PCE (Figure S3).

In addition, we have fabricated devices using the best combination of mixed PA stack (Me-4PACz and PHA) with high-performance triple halide triple cation absorbers passivated with a piperazinium salt to show the highest achievable performance with minimized bulk and ETL interface losses (see Figure S6). These devices show a very tight distribution of FF and *V*_OC_ statistics (84.3% average FF and 1.235 V average *V*_OC_) (Figure 2c), and the champion single junction devices (with 120 nm MgF_*x*_ antireflective coating on the glass side) achieved 22.2% PCE, with FF’s > 85% and *V*_OC_’s above 1.22 V. At a band gap of 1.65 eV, these results approach the radiative limit (*V*_oc-rad_ = 1.36 V, FF = 90.7%^33^), highlighting the potential of the developed HTL stack.

### 2.3 Charge Extraction

**Figure 3:**
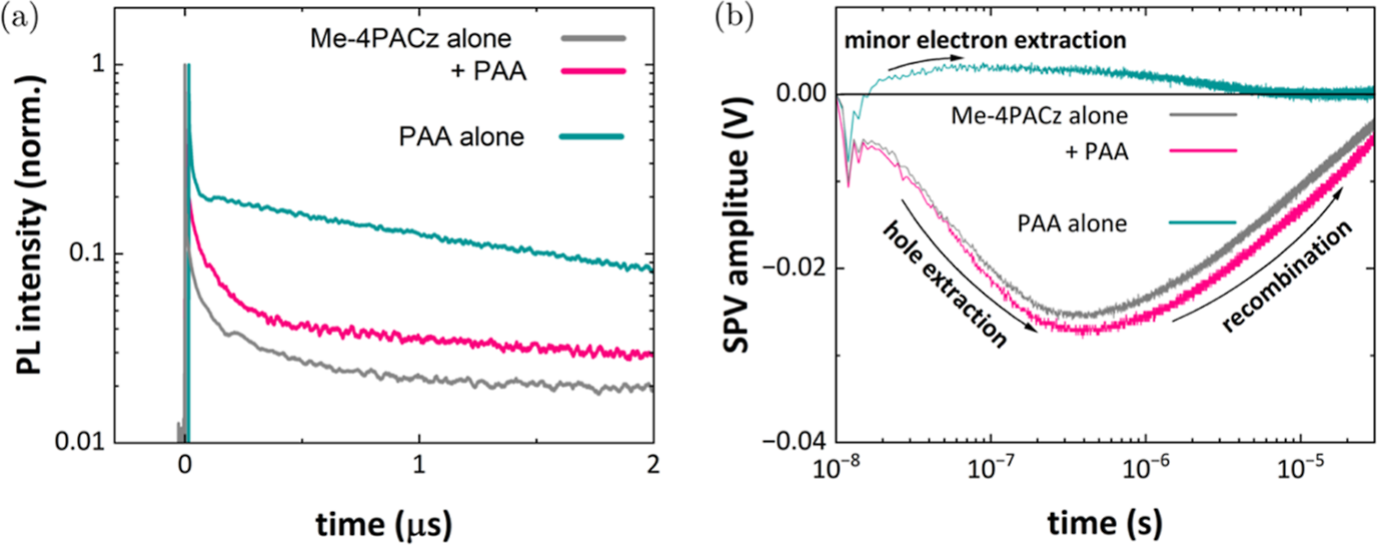
(a) Normalized tr-PL and (b) tr-SPV transients of Me-4PACz, the two-step approach with PAA, and PAA alone measured on glass/ITO/HTL/perovskite substrates using a 515 nm laser at a repetition frequency of 20 kHz.

We have investigated the dynamics of photogenerated charge carriers in ITO/HTL/perovskite stacks. We measured the transient photoluminescence (tr-PL) and transient surface photovoltage (tr-SPV) upon pulsed light excitation. The sign of the tr-SPV signal indicates the direction of charge separation. In the chosen configuration, a negative SPV signal marks the accumulation of electrons near the surface of the sample, for example due to the extraction of holes to the HTL.^29^ Both tr-PL and tr-SPV measurements were carried out under identical conditions at a repetition rate of *f*_rep_ = 20 kHz at ∼0.1 suns equivalent initial carrier concentration to allow a more accurate interpretation of the charge carrier dynamics.^29^Figure 3 shows the comparison between the half cells with Me-4PACz alone, with PAA alone, and a mixture of both.

The samples with Me-4PACz or the blend with PAA show a negative SPV signal, which corresponds to a hole-selective charge transfer through these materials into the ITO and is in agreement with previous tr-SPV measurements on Me-4PACz.^29^ These SPV-transients reach their maximum amplitude after ∼400 ns, which denotes the transition from the extraction to the recombination regime. In contrast, samples prepared using PAA alone show a completely different SPV-transient, with a persistent, significantly smaller positive signal. This behavior indicates an electron movement toward the ITO and can be interpreted either as trapping at the ITO/PAA/perovskite interfaces or even as a slight electron selectivity of the PAA alone. Either way, PAA by itself does not seem to be an efficient hole-selective transport layer. A similar behavior can be observed in the tr-PL (Figure 3b). Me-4PACz, or the mixture with PAA, shows a much more pronounced decay than the PAA only during the first ∼400 ns. In line with the tr-SPV results, this faster decay can be interpreted as quenching by efficient hole extraction. At later times, the tr-PL decays slower for Me-4PACz or the mixture with PAA decay, which can be attributed to lower interfacial recombination.

In addition, the SAMs deposition sequence was varied by omitting annealing and by additional washing in between the Me-4PACz and the PAA deposition to remove more Me-4PACz and attach more PAA to the ITO, as indicated by the contact angle measurements. We find that the more PAA is deposited on the surface, the less pronounced is the initial PL decay (Figure S8b), the slower the negative SPV signals build up, and the smaller the SPV amplitude is (Figure S8a). All these behaviors indicate poorer hole extraction and the benefit of the intermediate annealing step.

### 2.4 Perovskite/Silicon Tandem Solar Cells

**Figure 4:**
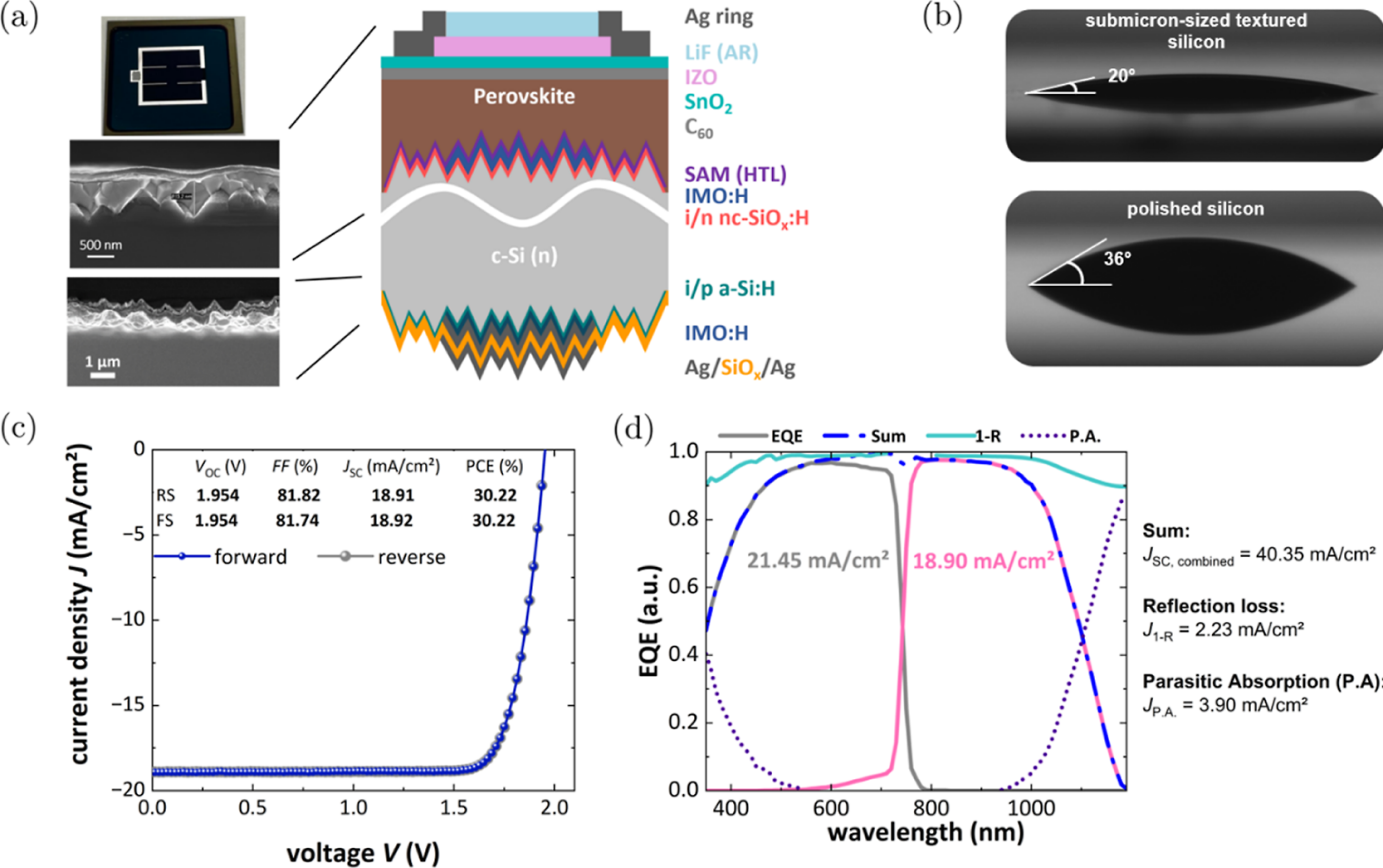
(a) Schematic illustration of the perovskite/silicon tandem solar cell based on 140 μm Cz-Si; (b) contact angle measurements on SHJ bottom cells with a submicron-sized textured or polished front side coated with mixed PA; (c) champion perovskite/silicon tandem *JV* curves—forward scan (*J*_SC_ to *V*_OC_) and reverse scan (*V*_OC_ to *J*_SC_); (d) EQE spectra (measured without grid) of a tandem device along with the sum of the two subcells (blue), 1-R, and parasitic absorption (1-R-EQE) with their respective current losses. The summed *J*_SC_ is 40.35 mA/cm^2^, with a reflection loss of 2.23 mA/cm^2^, and parasitic absorption of 3.90 mA/cm^2^ in the measured range.

Finally, in order to improve the optoelectronic properties of the top cell in a tandem device and the processing yield, the two-step approach (exemplarily using PAA) for the HTL coating was applied, and a double-side submicron-sized textured SHJ bottom cell was used as the bottom cell (Figure 4a). The impact of the submicron texture on the wetting behavior of the perovskite solution was investigated with respect to polished bottom cells by contact angle measurements as shown in Figure 4b. A strong improvement in wetting can be seen on submicron textures most likely due to microscopic effects induced by the topography that reduces the SFE. The contact angle was only measurable at the very beginning, at *t* = 0 s, as the perovskite ink spreads directly over the entire surface. This improved wetting on submicron-sized texture underlines the process improvements with a higher yield of tandem devices.

To ensure full coverage of the pyramids for an improved yield, the thickness of the top absorber layers was increased by increasing the molarity of the perovskite solution in the range of 1.6–1.8 M and the perovskite spin-coating recipe was adjusted accordingly.^34^ Increasing the molarity beyond that yielded milky films suggesting that one of the salts exceeded their solubility limit in this solvent system.

Increasing the molarity increases the thickness of the perovskite film, leaving less light for transmission to the Si bottom cell. As a result, more ∼current is generated by the top cell and less by the bottom cell, as can also be seen from the EQE measurements shown in Figure S9a. This shift in photocurrent contribution from the top cell to the bottom cell limits the short-circuit current density of respective tandem cells with high molarity. It leads to an increase in FF, which is explained by two effects. In a monolithically interconnected multijunction solar cell, the FF characteristics of the limiting cell become more pronounced under current mismatch conditions.^35^ Depending on the pseudo-FF of each subcell (which is a combination of the recombination and shunt characteristics of the subcell), the current mismatch can result in higher FFs than in a fully current matched scenario. In our devices, we observe increasing FFs as the current mismatch shifts toward the c-Si subcell (c-Si cell becomes limiting). FF characteristics of similar devices with the same top cell but a slightly different bottom cell can be found in our previous work.^2^ Additionally, the *V*_OC_ increases strongly (Figure S9b), also indicating less (micro)shunting of the topcell at higher molarity. This could also contribute to the observed FF increase. These overall adjustments to the top-cell processing increase the performance of the tandem cells, resulting in efficiencies ≫27%.

Having optimized the perovskite absorber fabrication for submicron textures, proof-of-concept tandems with the initial developments of the sequential application spin–spin-anneal of Me-4PACz with PAA enabled a champion device with a PCE of 30.22% on a double-sided submicron-sized textured Cz-Si SHJ bottom cell achieving *V*_OC_’s up to 1.954 V and a stabilized PCE of 30.15%—Figure S10. The EQE presented in Figure 4d shows that the two subcells are not in current matching conditions, being strongly silicon-limited (*J*_top_ – *J*_bottom_ = 2.6 mA/cm^2^). Notably this mismatch is in part the explanation for the extremely low hysteresis as pointed out by Messmer et al.^36^ Overall, we demonstrate a high total short-circuit current density of 40.35 mA/cm^2^ (sum of top and bottom cell values), which is remarkable considering the use of an industrially relevant silicon bottom cell with a thickness of only 140 μm and one of the highest reported for thin Cz material.

These results demonstrate the potential of submicron-sized textured Cz-silicon bottom cells for industry-compatible tandem cells with solution-processed perovskite top cells to achieve high efficiencies. Furthermore, our work demonstrates a simple, implementable solution to circumvent the film formation issue associated with Me-4PACz while maintaining a high charge extraction rate and good passivation properties. The current work represents a preliminary exploration of the combination of the two molecules in the HTL, with the more promising process variation, such as the sequential spin coating with an intermediate annealing step with PHA and TH + PCl, to be explored in future studies.

## 3 Conclusion

In this work, we present a sequential application of small molecules based on a PA structure and the hydrophobic Me-4PACz to improve the wettability of the perovskite precursor for solution-processing. The improved wetting enabled a higher yield due to lower shunt fractions in single-junction perovskite solar cells. The newly developed HTL stack (combination of Me-4PACz and PA) does not hinder hole extraction as confirmed by tr-PL and tr-SPV measurements. The investigation of the effect of the chain length of the spacer of the small PAs showed an improvement in the device performance (higher average *V*_OC_) with increasing chain length (PAA–PPA–PHA). With the presented the HTL combinations (triple cation triple halide absorbers), single junction device performance above 22% was achieved with wide bandgap (1.65 eV) perovskite absorbers. By integrating the improved HTL stack into a perovskite/silicon tandem solar cell based on industrial (140 μm thick) Cz double-sided submicron textured SHJ bottom cells, we have demonstrated efficiencies >30%. This paves the way for more efficient, high production yield, industrially relevant, perovskite/silicon tandem solar cells.

## 4 Methods

### 4.1 Perovskite Fabrication and Deposition

#### 4.1.1 Triple Cation Absorber

The triple cation perovskite (TCP) absorber used in this work is Cs_0.05_(FA_0.90_MA_0.10_)_0.95_Pb(I_0.80_Br_0.20_)_3_. The absorber is prepared by the one-step antisolvent method. Three separate stock solutions are prepared. The first consists of 1.65 M FAI (Dyenamo, >99.99%) and 1.8 M PbI_2_ (TCI, >99.99%) in 4:1 (volume ratio) DMF/DMSO. The second consists of 0.825 M MABr (Dyenamo, >99.99%), 0.825 M FABr (Dyenamo, >99.99%), 1.8 M PbBr_2_ (TCI, >99.99%) in 4:1 (volume ratio) DMF/DMSO. The third is 1.5 M CsI (Alfa Aesar, >99.9%) in DMSO. These solutions are mixed in a volume ratio of 800:200:40, respectively, to obtain the TCP (1.65 eV) precursor. With 10 s left in the spin-program, 300 μL of anisole (Sigma-Aldrich, >99%) is dropped onto the substrate. Five mM of pFBPA (Abcr, >95%) is subsequently added for optimal performance. The samples are annealed at 100 °C for 15–20 min inside the glovebox. For single junction devices, 1.5 M solutions are used instead of 1.8 M. For the triple halide triple cation absorbers, an additional 10 mg/mL of PbCl_2_ and 3.5 mg/mL MACl precursors are added to the triple cation double halide mixture. Here the pFBPA additive is not used. For the PCl (>99%, Dyenemo) passivation, 0.1 mg/mL in IPA solution is used.

#### 4.1.2 Fabrication of Single-Junction Perovskite Solar Cells

0.7 mm ITO (15 Ω/square—Kintec) coated glass substrates are cleaned with Helmanexx, DI, acetone, and IPA, respectively, in an ultrasonic bath for 15 min. Immediately prior to SAM deposition, the samples are exposed to UV–ozone for 15 min. The Me-4PACz solution (1 mg/mL) is spin-coated on substrates for 100 μL with a program of 10 s resting time, 3000 rpm, 300 rpm/s. After the first 10 s of spin-coating, a further 100 μL of SAM solution is dynamically coated onto the substrates. Then the substrates are annealed for 10 min at 100 °C. For the deposition of the perovskite absorber, the triple cation solution (100 μL) is spread on the substrate (2.5 × 2.5 cm^2^) and then spin-coated at 3500 rpm with 2000 rpm/s for 35 s. For the PCl passivation, 0.1 mg/mL solution is spin-coated at 6000 rpm (dynamic spin-coating) for 30 s and annealed at 100 °C degrees for 10 min. Twenty nm of C_60_ (NanoC, >99.9%) is deposited by thermal evaporation with a base starting pressure of around 10^–6^ mbar at a rate of 0.2 A/s. Then the atomic-layer deposition (ALD)-SnO_*x*_ deposition is carried out at 100 °C with pulse purge times of 0.3/6/0.1/6 s for 220 cycles with TDMASn and H_2_O sources. The thicknesses used for the baseline devices are 25 nm measured with an ellipsometer on c-Si. For the metal contact, 130 nm of Ag is deposited by thermal evaporation at a rate of 1.5–2 A/s. The active area of the device is 0.2 cm^2^.

#### 4.1.3 Fabrication of Perovskite/Silicon Tandem Solar Cells

The bottom cells are based on ∼140 μm thick, n-type, Cz-grown as-cut 5″ Si wafers laser-cut to 4″. After an ozone rough cleaning, the wafers were submicron-textured by alkaline surface etching for 15 min in DI water, KOH, K_2_SiO_3_, and an ethylene glycol-based additive at 75 °C. Afterward the wafers were exposed to an RCA final cleaning (RCA1, HF, RCA2). The a-Si:H(i)/nc-SiOx:H(*n*) layer stack on the front and the a-Si:H(i)/a-Si:H(p) layer stack on the rear were deposited using plasma-enhanced chemical vapor deposition (PECVD) in an applied materials AKT1600 cluster tool. On the rear a layer stack of transparent conducting oxide (99% indium oxide from newSCOT target from ANP Corp.180 nm on flat)/Ag (400 nm on flat) was sputtered by DC sputtering and 20 nm (on flat) on the front, defining the cell area of ∼1 cm^2^ by aligned sputter masks on both sides. On the rear side, a 500 nm-thick SiO_*x*_ layer was deposited by PECVD over the full area (metallized and nonmetallized) and afterward an additional Ag layer was sputtered through sputter masks on the active area with a slightly larger contact area (1.3 × 1.3 cm^2^). After sputtering the samples were annealed on a hot plate at 210 °C for 10 min for 30 min in an oven in ambient air. The 4″ wafers were then laser-cut to 2.5 × 2.5 cm^2^ bottom cells.

The perovskite films were fabricated similarly to the top cell presented in.^2^ The HTL (SAM) was spin-coated (3000 rpm, 30 s) and annealed at 100 °C for 10 min. The wide bandgap (1.67 eV) TCP absorber [Cs_0.05_(FA_0.90_MA_0.10_)_0.95_Pb(I_0.80_Br_0.20_)_3_] was spin-coated with a molarity of (1.6, 1.7, or 1.8 M) to obtain thicker films and annealed at 100 °C for 20 min. After the perovskite spin-coating, a 15 nm thick C_60_ electron transport layer was thermally evaporated. Afterward, a 25 nm SnO_2_ buffer layer was deposited by thermal ALD. A 35 nm thick IZO layer was RF sputtered through a shadow mask and 500 nm Ag was deposited by thermal evaporation through a shadow mask (60 μm wide fingers, 3.6 mm space in between fingers, 750 μm wide busbar surrounding the active area). A low-temperature Ag paste was applied using a hand brush with a pad area of ∼1 mm^2^ for device measurements and annealed at 70 °C for 10 min on a hot plate under ambient air. Lastly, LiF_*x*_ (100 nm) was deposited as an antireflection (AR) coating over the full area by thermal evaporation.

### 4.2 Characterization

#### 4.2.1 JV

The 4PP measurement method is used. JV-measurements were made using a two-lamp (Halogen and Xenon) class AAA WACOM sun simulator with an AM1.5G irradiance spectrum at 1.000 W/m^2^. 0.1 cm^2^ shadows masks were used to measure cells with an area of 0.25 cm^2^ unless otherwise stated. Opaque devices are illuminated from the glass side. The single junction cells are measured at a scan rate of ∼0.20 V/s. Three-point weight MPP measurements are performed using an in-house written LabVIEW code.

For the measurement of tandem devices, before each measurement, the calibration of the AAA WACOM system is checked with three different certified cells of different spectral responses to minimize the spectral mismatch of the sources. The 1.21 cm^2^ cells are then measured at a scan rate of ∼0.1 V/s using a similar MPP tracking algorithm as for the single junction perovskite solar cells. For the measurement, a temperature-controlled (25 °C) brass chuck was used.

#### 4.2.2 EQE

EQE spectra were measured with a custom-made spectral response setup where the samples were irradiated with chopped light at a frequency of 217 Hz and the response was measured using a lock-in amplifier. For tandem cells, IR and blue light biases are used to saturate complementary subcells and to measure each subcell near short-circuit conditions. 0.7 and 1.2 V bias voltages are applied to the cell when measuring top and bottom cells, respectively. The spot size for EQE measurements is small enough to be measured without any illumination in the Ag grid. Hence, EQE spectra do not account for shading losses.

#### 4.2.3 UV VIS Measurements

To measure the reflectance spectrum a PerkinElmer Lambda 950 setup was used equipped with an integrating sphere. A black shadow mask with a circular opening was used, whereby the Ag metal fingers were still partially within the measurement area. To compensate for the reflectance losses caused by the Ag grid, the area metal shadowing was calculated (2.5%) and the reflectance spectrum was adjusted accordingly.

#### 4.2.4 SEM Measurements

Images were acquired with acceleration voltages ranging from 1 to 5 kV using an in-lens detector (Zeiss NVision 40).

#### 4.2.5 Steady-State PLQY Measurements

To measure in situ PL and PLQY, a custom-built steady-state PL setup is used. The light from the laser diode is coupled into a fiber directed into the integrating sphere and the sample is illuminated. Then the emission from the sample is homogenized by multiple reflections within the integrating sphere and coupled out into another fiber that is connected to a spectrometer. As long as the emission and the excitation spectra are different, in our case 750–800 nm and 532 nm, respectively, a careful comparison of the peaks allows to measure PLQY (down to 10^–6^). An optical density filter wheel helps to change the light intensity between 0.01 and 3 sun. As a calibration check, three fluorescent test samples with high specified PLQY (∼70%) supplied by Hamamatsu photonics were measured and the specified value could be accurately reproduced with a small relative error of less than 5%. Depending on the band gap of the absorber, the light intensity of the QFLS measurements is tuned to 1-sun, using an external c-Si photodetector.

#### 4.2.6 Transient Photoluminescence and Transient Surface Photovoltage Measurements

In this study, an examination of the impact of the additives on charge carrier extraction and nonradiative recombination losses occurring at the interface between a perovskite layer and a HTL was conducted. This investigation used a combination of tr-SPV and time-resolved photoluminescence (tr-PL) techniques, both performed under identical photogeneration conditions. Tr-PL, a widely utilized method for assessing charge carrier recombination in semiconductors, was complemented by tr-SPV, which probes carrier dynamics at buried interfaces, thereby enabling the distinction between charge carrier extraction (manifesting as the rise of SPV) and recombination (evident as the decay of SPV). Additionally, the direction of charge carrier movement was identified based on the voltage sign.^29^ In both experiments the samples were excited using a femtosecond laser (Menlo BlueCut) with a wavelength of 515 nm and a repetition rate of 20 kHz. The laser power was attenuated to 24 pJ/cm^2^/pulse, corresponding to a photoexcitation of 6 × 10^10^ photons/cm^2^/pulse. For a sample thickness of 500 nm, this resulted in a charge carrier generation of 2 × 10^14^ carriers/cm^3^, a concentration typical for perovskite thin films under 0.1 sun illumination to open circuit conditions. Photoluminescence emission was captured by time-correlated single photon counting with a PicoHarp300. A Geiger-mode avalanche photodiode was employed, accompanied by a 530 nm long-pass filter to suppress the 515 nm laser light. The tr-SPV signal was conducted in a parallel plate capacitor configuration, where the capacitor was formed between the reference electrode (a quartz cylinder partially coated with SnO_2_/F) and the sample electrode (ITO of the device). The signal was recorded through a high-impedance buffer connected to an oscilloscope.^29^ The encapsulation layer on top of the perovskite served as the capacitor insulator, augmenting the voltage without the need for additional insulators such as mica sheets.

#### 4.2.7 Contact Angle Measurements

A Kruss DSA 100 contact angle setup was used at ambient conditions (23 °C, 38%rh). The contact angle of water, diiodomethane and perovskite on the different surfaces have been measured optically and analyzed by the method of Owens–Wendt–Rabel and Kaelble to calculate their SFE.^30,31^

## Supporting Information Available

SHJ characteristics, formation procedure JV statistics, complete wetting envelope set, single-junction EQE, complete TRPL & trSPV transients set, concentration optimization, MPP tracking of tandem device, additional PLQY measurements (PDF)
