# Supplementary material for: Perovskite/Silicon Tandem Solar Cells Above 30% Conversion Efficiency on Submicron-Sized Textured Czochralski-Silicon Bottom Cells with Improved Hole-Transport Layers
Source: ACS Appl Mater Interfaces. 2024 Oct 30;16(45):62817–26. doi: 10.1021/acsami.4c09264 (PMC11565564; doi:10.1021/acsami.4c09264)
Supplement: Supplementary file 1 — am4c09264_si_001.pdf [file am4c09264_si_001.pdf]

Supporting information:

Perovskite/silicon tandem solar cells above 30% conversion efficiency on submicron-sized textured Cz-silicon bottom cells with improved hole-transport layers

Angelika Harter[1*$], Kerem Artuk[2$], Florian Mathies[1], Orestis Karalis[1], Hannes Hempel[1], Amran Al-Ashouri[1], Steve Albrecht[1], Rutger Schlatmann[1], Christophe Ballif[2,3], Bernd Stannowski[1], and Christian M. Wolff*[2]

[$] Authors contributed equally

* Corresponding authors: angelika.harter@helmholtz-berlin.de , christian.wolff@epfl.ch

[1]Helmholtz Zentrum Berlin (HZB), Solar Energy Department, Schwarzschildstraße 3, 12489 Berlin, 12489 Berlin, Germany

[2]École Polytechnique Fédérale de Lausanne (EPFL), Institute of Electrical and Microengineering (IEM), Photovoltaics and Thin-Film Electronics Laboratory (PV-Lab), Rue de la Maladière 71b, 2000, Neuchâtel, Switzerland

[3]CSEM, Sustainable Energy Center, Rue Jaquet-Droz 1, 2000, Neuchâtel, Switzerland

Additional Figures and Tables:

Table S1: *JV* characteristics of perovksite/silicon tandem solar cells based on SHJ bottom cells using CZ material.

Year	Institution	Top cell	Cz wafer thickness (µm)	Area (cm²)	V_{OC} (V)	*FF* (%)	J_{SC} (%)	PCE (%)	Ref
2021	HZB	2PACZ/ $Cs_{0.05}(MA_{0.23}FA_{0.77})_{0.95}Pb(Br_{0.23}I_{0.77})$	100	1.00	1.93	81.05	17.8	27.8	[13]
2023	Kaneka	SAM/ $Cs_{0.05}(FA_{0.74}MA_{0.26})_{0.95}Pb(I_{0.74}Br_{0.26})_3$	145	0.15	1.93	77.87	19.5	29.2	[14]

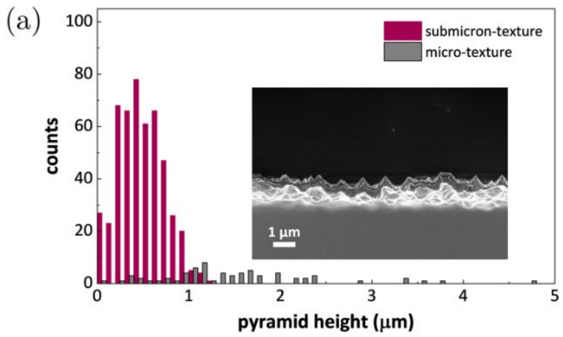

(c)

Sample	J_{SC} (mA/cm²)	V_{OC} (mV)	FF (%)	PCE (%)
Submicron, LONGi	39.25	739.0	81.99	23.78
Submicron, NorSun	39.15	743.7	80.26	23.37
Micro, NorSun	39.05	742.5	80.58	23.36

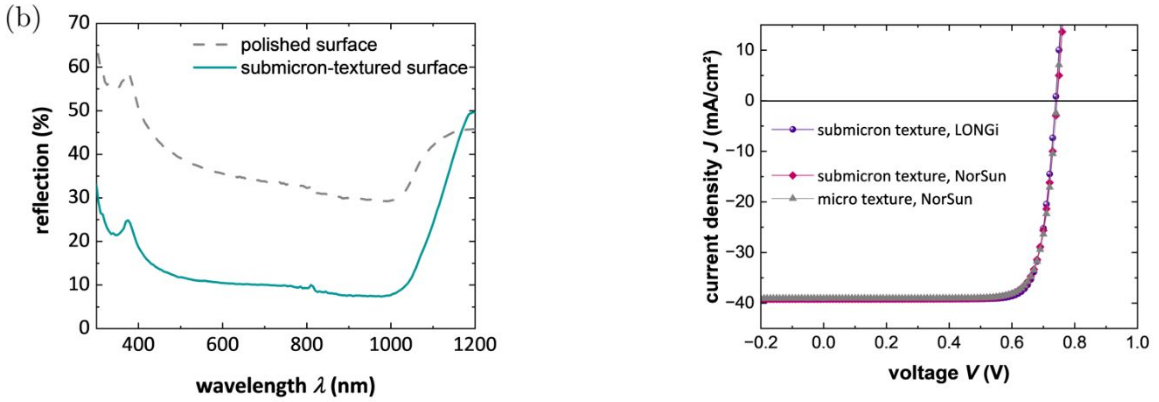

Fig. S1: Characteristics of the submicron texture: (a) Height distribution compared to the micro textured sample with a cross section of the rear side of the bottom cell as inlet; (b) Reflectivity measured on the wafer with the submicron texture compared to a polished surface, commonly used in bottom cells; (c) *JV* curves and characteristic parameters of SHJ solar cells with the submicron texture on NorSun material compared to our micro textured surface, as well as on LONGi as used in the bottom cell of this work.

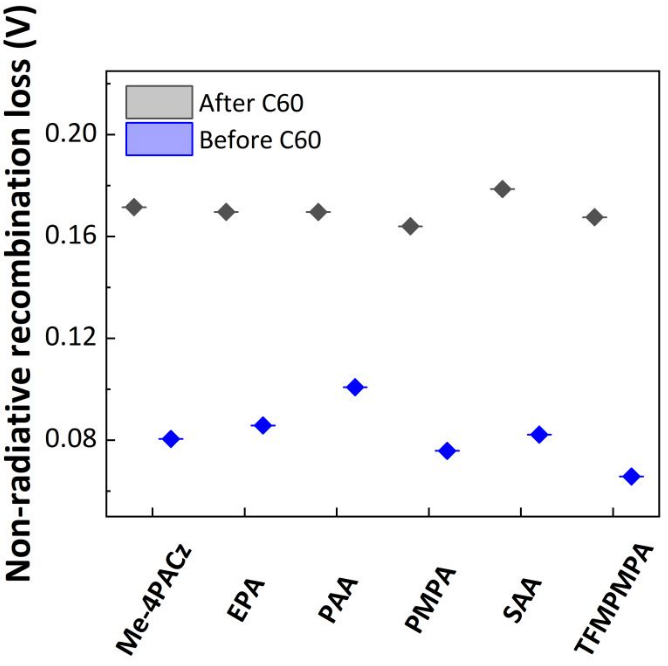

Fig. S2: Non-radiative recombination loss ($qV_{\text{non-rad}}$) extracted by PLQY measurements of the different combinations of PAs and Me-4PACz measured on a stack of glass/ITO/HTL/perovskite stack before and after C_{60} deposition.

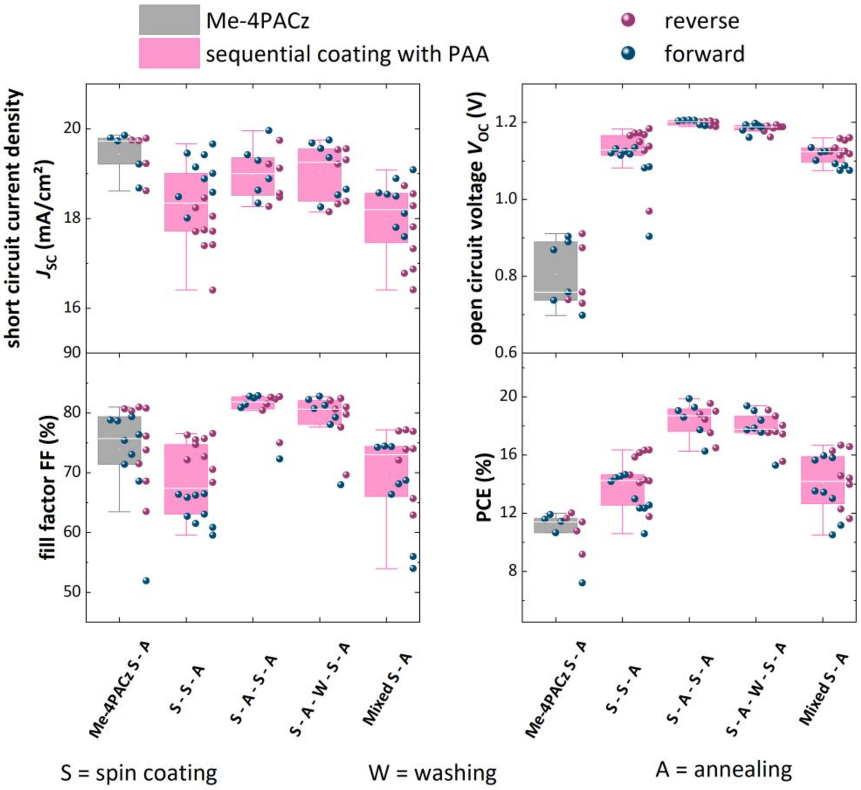

Fig. S3: Single junction solar cell performance for the combination of PAA and Me-4PACz measured under simulated AM 1.5G illumination, S=spin coating, A= annealing, W= washing.

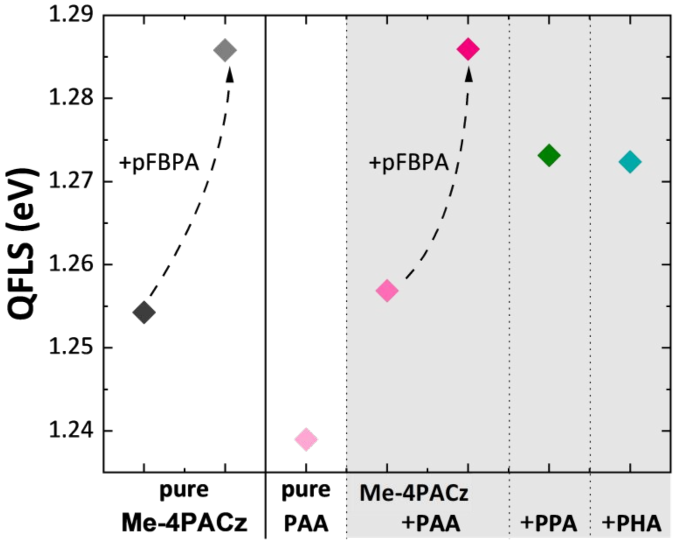

Fig. S4: QFLS of the different combinations of PAs and Me-4PACz measured on a stack of glass/ITO/HTL/perovskite using PAA; PPA, PHA as fillers. In addition, the effect of the mixture of pFBPA with the 3CAT perovskite solution on the QFLS splitting is shown.

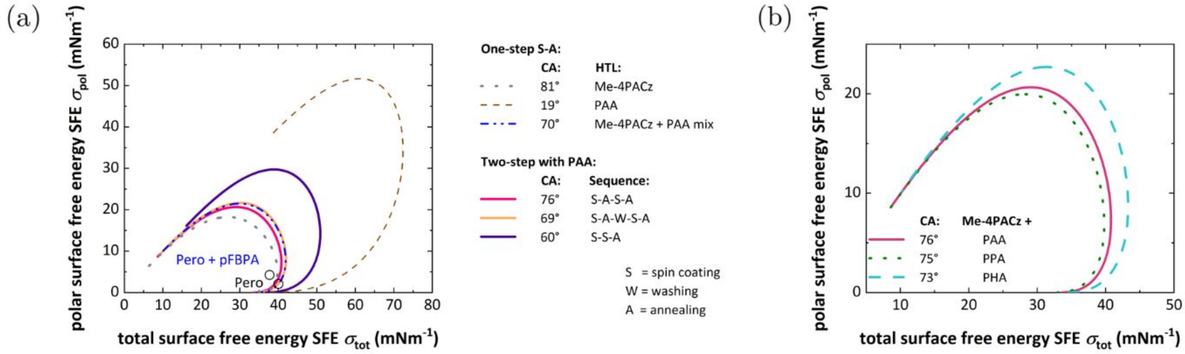

Fig. S5: Wetting envelope with the surface free energy (SFE) measured on glass/ITO/HTL samples **(a)** of different coating approaches using only Me4-PACz or PAA in a one-step approach (S-A), a mixture of Me-4PACz and PAA, and the sequential two-step approaches: S-A-S-A: coating Me4-PACz and annealing, then coating PAA and annealing. S-A-W-S-A: like S-A-S-A with EtOH washing in between. S-S-A. Coating Me4-PACz, coating PAA and annealing; Circles show the surface free energy measured of the perovskite inks with and without pFBPA, respectively; **(b)** of the two-step approach S-A-S-A spin coating first Me-4PACz and subsequently PAA, PPA, or PHA as filler molecules.

Table S2: Total surface free energy (SFE) measured on glass/ITO/HTL samples spin-coated with Me-4PACz alone, PAA alone and sequentially with PAA, PPA and PHA determined by CA measurements.

PA derivative	spin-coating sequence	total surface free enegery SFE σ_{tot} (mNm^{-1})		
		1-step	2-step (1. Me-4PACz; 2. PA)	2-step (1. PA; 2. Me-4PACz)
PAA	S - A only	72.4		
Me-4PACz	S - A only	39.8		
PAA	S - A - S - A		40.8	55.8
PAA	S - A - W - S - A		42.0	50.0
PAA	S - S - A		50.9	45.1
PAA	Mix S - A	41.9		
PPA	S - A - S - A		39.8	66.5
PPA	S - A - W - S - A		43.5	64.4
PHA	S - A - S - A		43.3	53.6
PHA	S - A - W - S - A		44.0	51.3

Table S3: Dispersive surface free energy (SFE) measured on glass/ITO/HTL samples spin-coated with Me-4PACz alone, PAA alone and sequentially with PAA, PPA and PHA determined by CA measurements.

PA derivative	spin-coating sequence	dispersive surface free enegery SFE σ_{dis} (mNm^{-1})		
		1-step	2-step (1. Me-4PACz; 2. PA)	2-step (1. PA; 2. Me-4PACz)
PAA	S - A only	38.0		
Me-4PACz	S - A only	34.8		
PAA	S - A - S - A		33.5	35.1
PAA	S - A - W - S - A		34.0	36.5
PAA	S - S - A		36.7	39.0
PAA	Mix S - A	34.1		
PPA	S - A - S - A		32.9	36.3
PPA	S - A - W - S - A		33.5	36.0
PHA	S - A - S - A		34.6	40.9
PHA	S - A - W - S - A		34.3	39.7

Table S4: Polar surface free energy (SFE) measured on glass/ITO/HTL samples spin-coated with Me-4PACz alone, PAA alone and sequentially with PAA, PPA and PHA determined by CA measurements.

PA derivative	spin-coating sequence	polar surface free enegery SFE σ_{pol} (mNm^{-1})		
		1-step	2-step (1. Me-4PACz; 2. PA)	2-step (1. PA; 2. Me-4PACz)
PAA	S - A only	34.4		
Me-4PACz	S - A only	5.00		
PAA	S - A - S - A		7.30	20.7
PAA	S - A - W - S - A		8.00	13.5
PAA	S - S - A		14.2	6.10
PAA	Mix S - A	7.80		
PPA	S - A - S - A		6.90	30.2
PPA	S - A - W - S - A		10.0	28.4
PHA	S - A - S - A		8.70	12.7
PHA	S - A - W - S - A		9.70	11.5

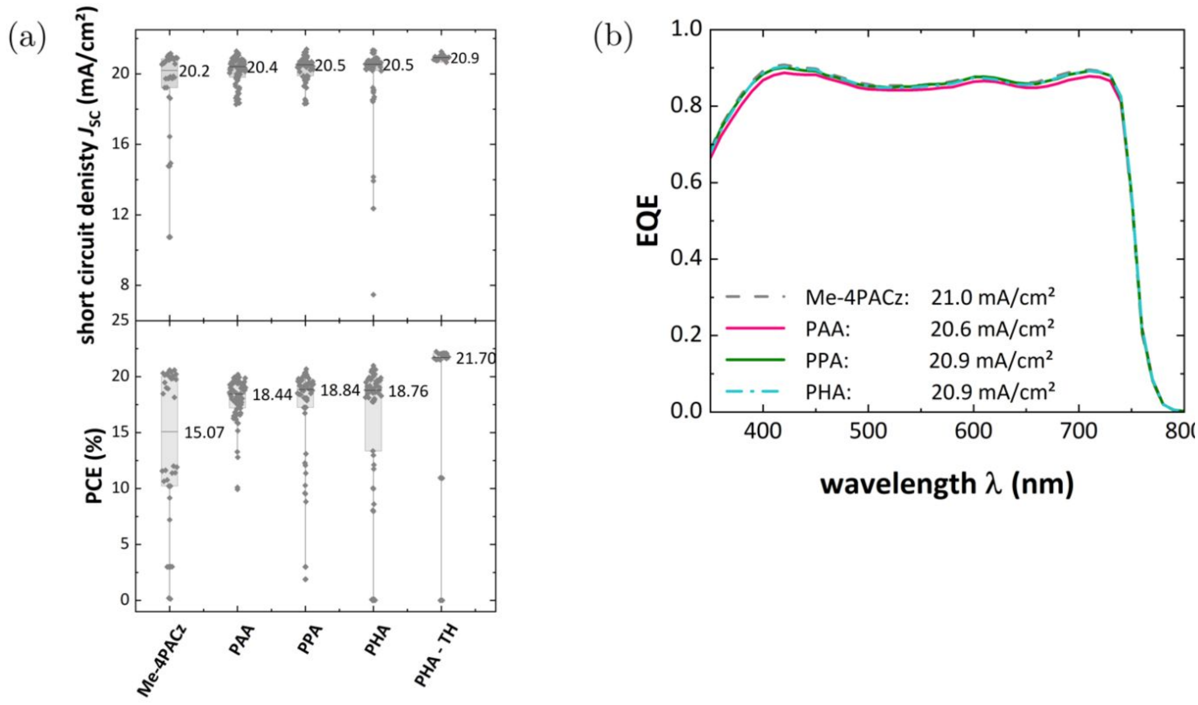

Fig. S6: Performance of single junction solar cells for the sequential spin-coating with Me-4PACz and a PA derivative: **(a)** JV data measured under simulated AM 1.5G illumination; **(b)** External quantum efficiency (EQE) spectra for the combination of Me-4PACz and PAA, PPA and PHA;.

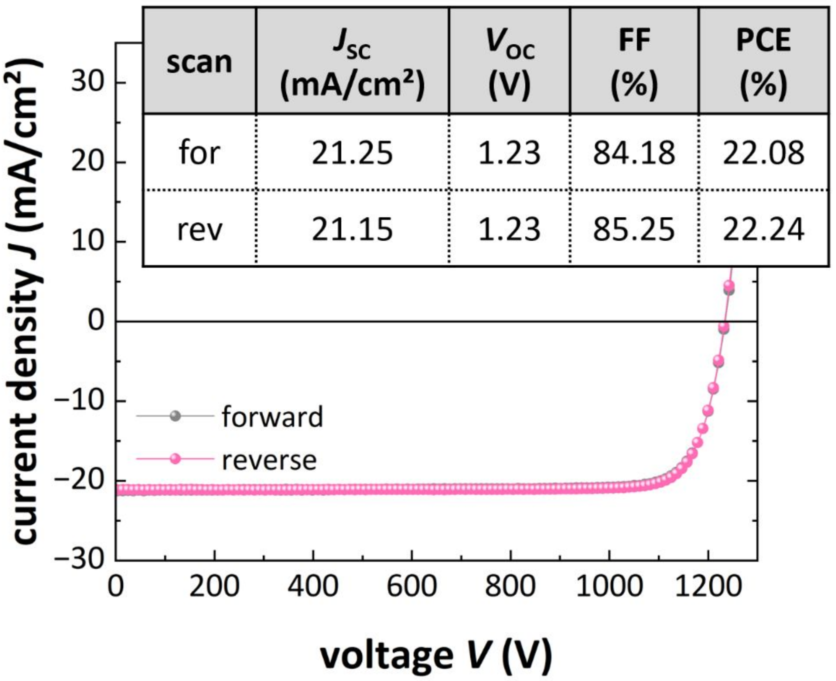

scan	J_{SC} (mA/cm²)	V_{OC} (V)	FF (%)	PCE (%)
for	21.25	1.23	84.18	22.08
rev	21.15	1.23	85.25	22.24

Fig. S7: Champion single junction devices (triple halide triple cation perovskite; with 120 nm MgF_x anti-reflective coating on the glass-side): *JV* curves under simulated AM 1.5G illumination at a scan rate of 200 mV s^{-1} in forward (J_{SC} to V_{OC}) and reverse scan (V_{OC} to J_{SC}) with Me-4PACz + PHA as HTL.

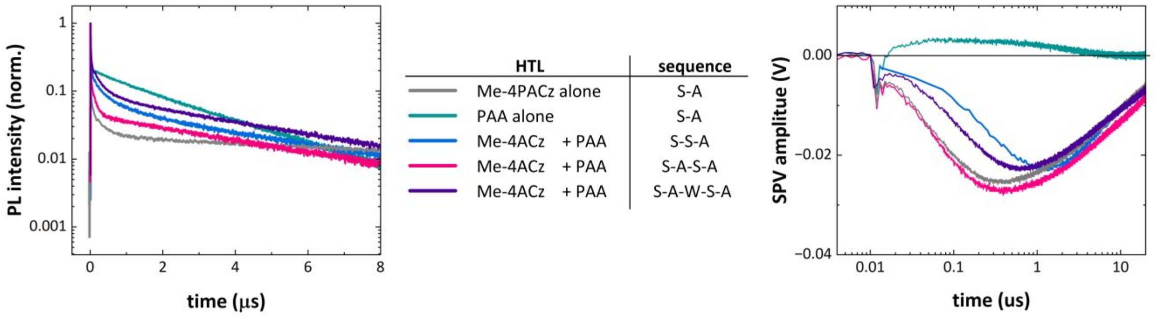

Fig. S8: Normalized transients of (left) tr-PL and (right) tr-SPV for samples spin-coated with Me-4PACz alone, PAA alone and the sequential spin-coating with different sequences using PAA. Tr-PL and tr-SPV was measured on glass/ITO/HTL/perovskite substates using a 515 nm laser at a repetition frequency of 20 kHz.

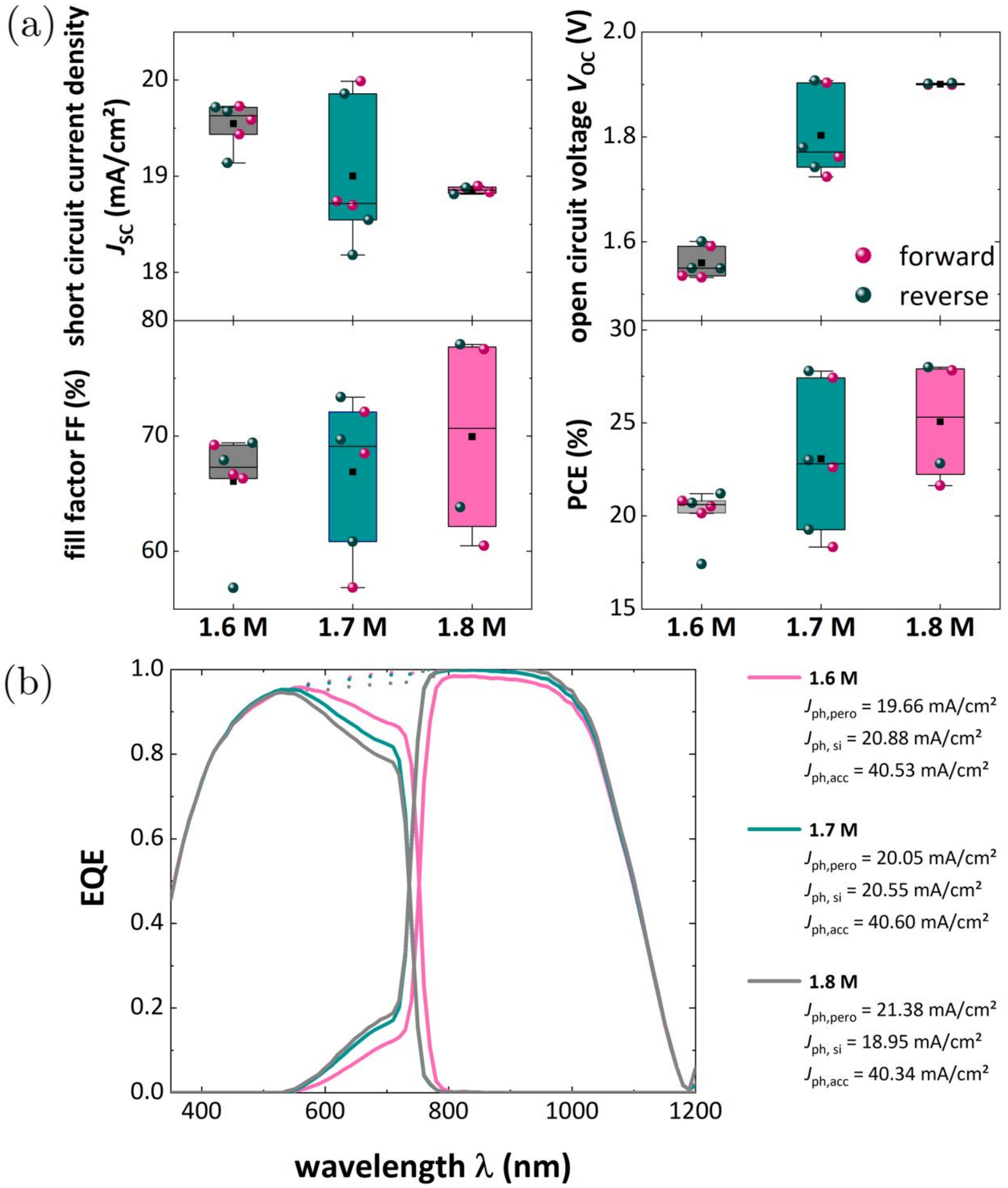

Fig. S9: Performance of the perovskite/silicon tandem solar cells spin-coated with perovskite in different molarities (1.6 M - 1.8 M): (a) *JV* curves– forward scan (J_{SC} to V_{OC}) and reverse scan (V_{OC} to J_{SC}) measured under simulated AM 1.5G illumination; (b) EQE spectra (measured without grid) of a tandem device with the sum of the two sub-cells (dashed).

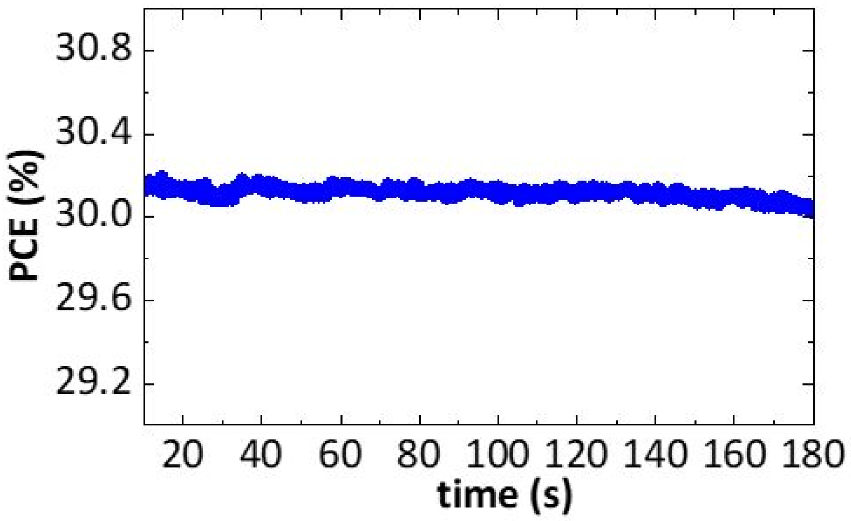

Fig. S10: Maximum power point tracking of the champion tandem device for 180 s stabilizing at 30.15%.

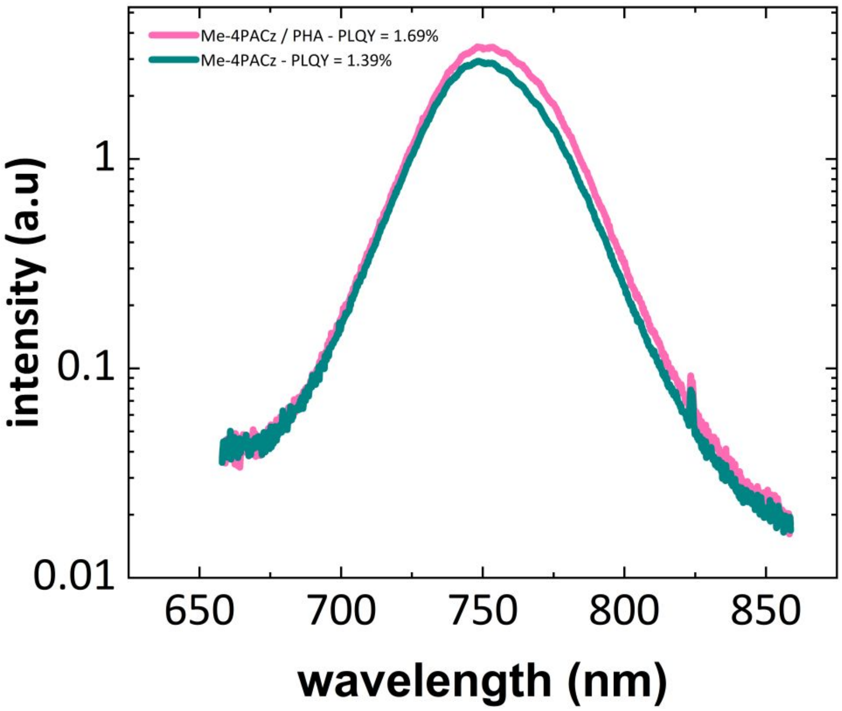

Fig. S11: PLQY measurements of Me-4PACz and Me-4PACz /PHA with PAA measured on a stack of glass/ITO/HTL/perovskite.
